# Mobility limitations related to reduced pulmonary function among aging people with chronic obstructive pulmonary disease

**DOI:** 10.1371/journal.pone.0196152

**Published:** 2018-05-01

**Authors:** Francesc Medina-Mirapeix, Roberto Bernabeu-Mora, Mª Piedad Sánchez-Martínez, Joaquina Montilla-Herrador, Myriam Bernabeu-Mora, Pilar Escolar-Reina

**Affiliations:** 1 Department of Physical Therapy, University of Murcia, Murcia, Spain; 2 Department of Pneumology, Hospital General Universitario J M Morales Meseguer, Murcia, Spain; 3 Department of Internal Medicine. Hospital Vega Baja de Orihuela, Alicante, Spain; National and Kapodistrian University of Athens, GREECE

## Abstract

**Background:**

Chronic obstructive pulmonary disease (COPD) is a major cause of disability. We aimed to analyse the impact of reduced pulmonary function on non-respiratory impairments and mobility activity limitations in an elderly population with COPD and to elucidate which specific limitations on mobility are related to reduced pulmonary function

**Methods:**

Cross-sectional study of 110 patients with COPD, recruited from public and university hospital. The effect of impaired pulmonary function on the risk of non-respiratory impairments and mobility limitations was analysed using validated measures, including: the 6-Minute Walk Test (6MWT), skeletal muscle strength, the Short Physical Performance Battery (SPPB), and self-reported mobility questionnaire. Multivariate analysis was used to control for confounders such as age, sex, height, education, and cigarette smoking.

**Results:**

Greater impairment of pulmonary function was associated with less distance walked during the 6MWT, poorer SPPB scores, and greater risk of self-reported mobility limitations (p<0.05). Lower forced expiratory volume in 1 s was also associated with a greater risk of limitations in carrying items under 10 pounds (4.54 kg), walking alone up and down a flight of stairs, and walking two or three neighbourhood blocks. There was no clear statistical relationship between pulmonary function impairment and skeletal muscle strength.

**Conclusions:**

Impaired pulmonary function was associated with the 6MWT score and limitations on performance-based and self-reported mobility activities, but not with skeletal muscle strength among elderly COPD patients.

## Introduction

As describe previously by Haraguchi et al [1], chronic obstructive pulmonary disease (COPD), one of the most prevalent health conditions, is the fourth leading cause of death worldwide. In the last decades, COPD morbidity and mortality have increased in many countries, in part due to an aging population world-wide. Despite medical advances in the management of COPD, the incidence of COPD-related disabilities has risen exponentially compared with a decline in other chronic diseases (such as cardiovascular diseases) [2]. Currently available pharmacological treatments based on the severity of airflow obstruction have minimal impact on disease progression; therefore, a strategy to curb the course of disability would have important clinical and public health implications [3].

As note by Eisner et al [3], the assessment of pulmonary function is the most important indicator of respiratory impairment in COPD, but paradoxically it is a poor predictor of disability in terms of daily activity limitations [3, 4]. In contrast, the development of non-respiratory impairments such as in muscle strength, dyspnoea, exercise performance, and mobility limitations have a greater impact on disablement [5]. According to previous studies, the impact of respiratory impairment on daily activity limitations could be mediated by its initial effect on non-respiratory problems and mobility activities. Specifically, Eisner et al. found that pulmonary function in patients with COPD was related to skeletal muscle strength, exercise performance, lower extremity functioning, and self-reported mobility limitations [3]. These author intentionally sampled non-elderly adults between 40 and 65 years old; thus, it is unclear if these associations are maintained in elderly patients with COPD.

The primary aim of this study was to analyse the impact of pulmonary impairment on non-respiratory deficiencies and limitations on mobility activities in an elderly population with COPD. We hypothesized that greater reduction in pulmonary function would lead to non-respiratory and mobility impairments in elderly COPD patients. A secondary aim was to elucidate which specific mobility activities are most affected by pulmonary function impairment.

## Materials and methods

### Study design and participants

This was a cross-sectional study. Patients with stable COPD were prospectively recruited from an outpatient pulmonary service at Public University Hospital, during 2015. All study participants provided written informed consent, and the study protocol was approved by the institutional review board of the hospital called the “Ethical Committee of Clinical Research of the General University Hospital” (approval number: EST-35/13).

Patients were included in this study if they fulfilled the following criteria: a diagnosis of COPD according to the Global Initiative for COPD (GOLD) recommendations (a forced expiratory volume in 1 s (FEV_1_)/forced vital capacity post-bronchodilator ratio of <70%) [6], in stable stage (without exacerbations in the previous 3 months) and aged between 60 and 80 years. Patients with an unstable cardiac condition within 4 months of the start of the study were excluded, as were those with cognitive deterioration, or who were unable to walk. Over a 1-year period, a consecutive sample of eligible patients was identified on a rolling-basis from patient health examinations. A pulmonary physician assessed their eligibility for inclusion.

### Measures

We collected sociodemographic and clinical characteristics, pulmonary function, non-respiratory impairments, and mobility limitations. Sociodemographic characteristics included age, sex, and educational level (primary/high school/college or graduate degree). The clinical variables included a history of cigarette smoking, grade of dyspnoea (measured by the modified British Medical Research Council [mMRC] scale) [7], COPD Assessment Test (CAT^TM^) [8], and frequency of exacerbations in the last year.

#### Pulmonary function

To assess pulmonary function, each patient underwent post-bronchodilator spirometry with a MasterScope Spirometer (version 4.6, Jaeger, Würzburg, Germany), according to the American Thoracic Society guidelines [9]. To calculate the percent of predicted pulmonary function values, we used predictive equations derived from the Third National Health and Nutrition Examination Survey [10].

#### Assessment of non-respiratory impairments

We measured skeletal muscle strength and the 6-minute walk test (6MWT). The skeletal muscle strength of the lower and upper extremities was assessed on the dominant side with a hand-held dynamometer (Nicholas Manual Muscle Tester, model 01160; Lafayette Instrument) and handgrip dynamometer (KERN MAP 80K1, KERN & Sohn GmbH 1), respectively. The testing positions used for assessing muscle strength were based on previous descriptions and on the guidelines provided in the dynamometer manufacturer’s manual [11, 12]. For the strength assessment of the quadriceps, participants remained seated on a raised plinth, and the examiner placed the participant’s knee in a flexed position at 70 degrees; resistance was applied by the examiner in the direction of flexion with the dynamometer placed on the anterior tibia, 5 cm above the lateral malleolus, and a break test was performed [13]. For the elbow flexor strength assessment, participants remained seated and kept his or her arm at the side, with the shoulder adducted and neutrally rotated, the elbow flexed at 90 degrees, and the forearm in supination. The assessor applied the resistance in the distal part of the forearm towards the ground and the break test was repeated [14].

As described previously by Eisner et al [3], power grip strength was measured with a handgrip dynamometer. In a seated position, each subject kept his or her arm at the side, with the shoulder adducted and neutrally rotated, the elbow flexed at 90 degrees, and the forearm in a neutral position between supination and pronation. The examiner stabilized the elbow, and the subject was asked to squeeze the dynamometer, exerting a maximum grip. All measures of knee, elbow, and handgrip strength were repeated two times for each patient, and the individual average was calculated and used for the analysis [15].

The 6MWT was performed indoors, along a flat, straight, 30 m walking course, supervised by two well-trained nurses (with a mean of 19 years of experience), according to American Thoracic Society guidelines [16]. Patients were instructed and encouraged to walk as far as possible in 6 min, using standard incentive phrases every minute. Patients were permitted to stop and rest during the test, but were instructed to resume walking as soon as they felt able to do so.

#### Assessment of mobility activities

We used the short physical performance battery (SPPB) according to the National Institute on Aging protocol [17] and a self-reported mobility questionnaire used by Sternfeld [18]. The SPPB was performed in the following sequence: a) standing balance tests, b) 4 m gait speed test, and c) five-repetition sit-to stand motion test. The standing balance portion requires participants to maintain, for 10 s each, stances with their feet placed side by side, semi-tandem, and in tandem. The scores ranged from 0 to 4 (maximum performance). The 4MGS measured the time needed to walk 4 m at a typical pace. The 5STS required participants to rise from a chair with their arms across their chest, five times. Scores from 1 to 4 were assigned based on the quartile of length of time to complete the task. A summary score integrated the three performance measures, ranging from 0 to 12 [17, 19]. Standardized instructions were given for each of the three SPPB components, and we used standardized equipment for all patients. For example, all sit-to-stand manoeuvres were performed from a chair, without armrests, with a seat positioned at a height of 43 cm and a depth of 47.5 cm [17].

The self-reported mobility questionnaire comprised ten items related to two mobility activity domains defined by the International Classification of Functioning, Disability, and Health (ICF) [20]. This included five items related to the “changing and maintaining body position domain” (stooping, crouching, or kneeling; standing in place for 15 min or longer; getting up from a stooping, crouching, or kneeling position; sitting for long periods; and standing up after sitting in a chair); and five items corresponded to the “walking and moving domain” (pushing objects like a living room chair; moving or carrying light objects under 10 lb or 4.54 kg; moving or carrying heavy objects over 10 lb; walking alone up and down a flight of stairs; and walking two to three neighbourhood blocks). The patients assessed the degree of difficulty in performing the activity, based on the following response options: none, a little, some, a lot, do not do it, on doctor’s orders, unable to do it, and never do that activity. Each item was classified as “having a limitation” when patients indicated that they had “some” or “a lot of difficulty” with one or more items, or when they were told by a doctor not to do something. Furthermore, the proportion of those activities with a limitation was calculated for each patient.

### Statistical analysis

Descriptive statistics were used for the sociodemographic and clinical characteristics of participants. We used linear regression analysis to assess the association between pulmonary function impairment (FEV_1_) and performance on the 6MWT, the SPPB, and the percentage of activities having a limitation according to the self-reported mobility questionnaire. In addition, this analysis was used to study the relationship between FEV_1_ and skeletal muscle strength tests. To examine potential confounders, we used two sets of analyses that controlled for covariates, as described previously by Eisner et al [3]: age, sex, and height and age, sex, height, educational level, and smoking status (current vs. former or never).

Previously, we used linear regression to evaluate model assumptions. The linearity assumption was analyzed using scatterplots of the data; also we compared the fitted regression line with a regression line fitted by a locally weighted regression scatterplot smoother (LOWESS) procedure [21]. Residual vs. fitted plots and residual vs. predicted plots were used to analyze the functional form and the constant variance assumption. We used Kolmogorov Smirnov test to evaluate the normality requirement. Boxplots were used to check for outliers. The assumptions were met for all linear models. We were performed tests for interactions to evaluate whether GOLD stage, dyspnoea, and quality of life (CAT) modified the association between pulmonary function impairment and non-respiratory impairments and mobility activities. There was no evidence of statistical interaction in any case, so we did not include such interactions in any of the final models presented. To analyse the relationship between FEV_1_ and self-reported functional limitations in the mobility questionnaire, we used logistic regression with each question of the survey described by Sternfeld. Goodness-of-fit for the models were assessed using methods described elsewhere [22]. We used the LOWESS procedure to plot the logit of each dependent variable versus FEV_1_.

## Results

### Characteristics of patients

The sample characteristics are shown in Table 1. The average age of the sample was 70 years; most of the subjects were male (90%) and 71,8% were former smokers who didn’t smoke at that moment. The majority of subjects were GOLD stage D. In addition, the mean percentage of mobility activities with limitations was 43.67%.

**Table 1:** Baseline characteristics of 110 adult patients with COPD.

Characteristic	n (%) mean (± SD)
**Sociodemographic**	
Age, mean (± SD)	70 (5.74)
Male, n (%)	99 (90)
Educational level, n (%)	
Primary studies	35 (32.1)
High school	44 (40.4)
University	30 (27.5)
**Clinical characteristics and pulmonary function**	
Smoking history, n (%)	
Current smoker	31 (28.2)
Former smoker	79 (71.8)
GOLD group, n (%)	
A	20 (18.2)
B	18 (16.4)
C	8 (7.3)
D	64 (58.2)
mMRC score, median (interquartile range)	1.00 (1.00)
CAT ≥10, n (%)	81 (73.6)
Exacerbations ≥ 2 in last year, n (%)	66 (60)
FEV_1_ (litres), mean (± SD)	1.27 (0.45)
FEV_1_ (% predicted), mean (± SD)	50.6 (16.3)
FEV_1_/FVC ratio, mean (± SD)	57.96 (8.27)
FEV_1_ decrease (liter), mean (± SD)	1.26 (0.49)
**Non-respiratory impairments and mobility**	
6MWT (m), mean (± SD)	340.28 (84.65)
Quadriceps strength (Kg), mean (± SD)	15.45 (2.87)
Elbow strength (Kg), mean (± SD)	14.65(3.06)
Grip strength (Kg), mean (± SD)	27.47(7.81)
SPPB (score range 0–12), mean (± SD)	9.32 (2.00)
% activities with limitations, mean (± SD)	43.67 (26.08)

Abbreviations: SD, standard deviation; GOLD, Global initiative for Chronic Obstructive Lung Disease; mMRC, modified British Medical Research Council; CAT, COPD Assessment Test; FEV1 = forced expiratory volume in 1 s; FVC, forced vital capacity; 6MWT, 6-minute walking test; SPPB = Short Physical Performance Battery.

### Relevance of pulmonary function impairment

Results of multivariate models for non-respiratory impairments and mobility activities are shown in Table 2. The mean change in scores on each test was similar using both sets of covariates (age, sex, and height vs. age, sex, height, educational level, and smoking status). A greater pulmonary function impairment, as shown by a lower FEV_1_, was associated with a lower performance in the 6MWT (per 1 liter (L) decrement in FEV_1_: mean score decrease -82.86 meters, 95% confidence interval (CI): -116.62, -49.11). In contrast, there was no clear statistical relationship between pulmonary function impairment and skeletal muscle strength in any of the muscle groups tested. In relation to mobility activities, a higher decrement in FEV_1_ was associated with a lower score on a performance-based battery (per 1 L decrement in FEV_1_: mean score decrease = -1.11, 95% CI: -1.98, -0.24) and a higher percentage of mobility activities with limitations (per 1 L decrement in FEV1: mean score decrease 13.24%, 95% CI: 0.15, 26.33).

**Table 2:** Relevance of FEV1 on non-respiratory impairments and mobility activities.

Tests	Mean change in score^a^	95% confidence interval	*p-*value	Mean change in score^b^	95% confidence interval	*p-*value
**Non- respiratory impairments**						
6-Minute Walk Test (m)^c^	-85.32	-117.31, -53.33	0.000	-82.86	-116.62, -49.11	0.000
Quadriceps (kg)	- 1.74	- 4.14, 0.66	0.15	- 1.96	- 4.51, 0.58	0.12
Elbow flexors (kg)	- 0.96	- 3.19, 1.25	0.39	- 0.67	- 3.03, 1.68	0.57
Grip (kg)	- 0.89	- 6.02, 4.23	0.73	- 0.87	- 6.29, 4.55	0.75
**Mobility activities**						
SPPB (range 0–12)^d^	-1.18	-2.00, -0.36	0.005	-1.11	-1.98, -0.24	0.01
Self-reported questionnaire (0–100)	16.14	4.28, 27.99	0.008	13.24	0.15, 26.33	0.047

^a^ Per 1 liter (L) decrease in forced expiratory volume in 1 s (FEV1) controlling for age, sex, and height.

^b^ Per 1 L decrease in FEV1 controlling for age, sex, height, educational level, and smoking.

^c^ Results from linear regression are the mean change in the dependent variable for each 1 L decrement in FEV1.

^d^ SPPB, Short Physical Performance Battery

Abbreviations: m, meters; kg, kilograms; SPPB, Short Physical Performance Battery

As shown in Table 3, a higher decrement of FEV_1_ was associated with a greater risk of limitation for three activities of the self-reported mobility questionnaire: moving or carrying items under 10 pounds (4.54 kg), walking alone up and down a flight of stairs, and walking two or three neighbourhood blocks.

**Table 3:** Odds ratios of specific mobility activities with limitations per 1 liter (L) decrease in forced expiratory volume in 1 s (FEV1).

Items from self-reported questionnaire	Odds ratio^a^	p value
Pushing objects	2.68	0.14
Stooping, kneeling	0.97	0.46
Getting up from a kneeling position	1.16	0.79
Carrying lighter objects	7.17	0.009
Carrying heavier objects	1.65	0.34
Standing >15 min	2.04	0.18
Sitting 1 h	0.98	0.98
Standing from a seated position	1.95	0.27
Walking up stairs	8.56	0.004
Walking in the neighbourhood	4.50	0.01

^a^ Controlling for age, sex, and height.

## Discussion

In this study, we examined the impact of impaired pulmonary function on the risk of non-respiratory impairments and mobility activity limitations among aging people with COPD. We found that impaired pulmonary function was related to the 6MWT and different levels of limitations on performance-based and self-reported mobility activities. Subjects with a higher decrement in FEV_1_ walked less distance in the 6MWT and had more mobility limitations.

Our findings about the negative relationship between FEV_1_ and the 6MWT and mobility activities are consistent with Eisner's results in younger patients. However, our study did not provide sufficient evidence on the relationship between FEV_1_ and skeletal muscle strength reported by Eisner [3]. One possible reason for this discordance may be that in older subjects’ strength is more determined by age-related sarcopenia than by lung function itself, as shown in the study by Morley et al., in which 5 to 50% of subjects over 60 years old had loss of skeletal muscle strength associated with age [23].

Our study also provided more information than the Eisner study [3]. Our results showed not all mobility activities were equally determined by pulmonary function. As expected, pulmonary function had a greater impact on activities that required greater energy consumption or oxygen (carrying lighter objects, walking up stairs, and walking in the neighbourhood). In contrast, we found another activity with high oxygen consumption (“carrying heavier objects”) was contradictory to our expectations. A possible reason is that heavier objects make this activity more dependent on the strength element than the pulmonary function element, as suggested in the previous paragraph [24]. Another possible reason is that patients give up, avoid, or limit this activity as a behavioural strategy used to manage disability [25].

The adjusted magnitude of the FEV_1_ effect on the set of activities of the SPPB (1.11 over a range of 12 points) and self-reported activities (13.2 over a range of 100) were similar in relative terms (around 10% of their range scores). Therefore, our study suggests that FEV_1_ independently affects, in a similar way, the mobility activities, whether they are measured with standardized or self-reported tests. Thus, both measures can be valid indicators of the effect of FEV_1_ on the capacity to perform important mobility activities in daily living [26].

Our study also found that in COPD patients aged between 60 and 80 years, a decrease of 1 L in FEV_1_ leads to a reduction of 80 m in the 6MWT. Our findings about the negative influence of FEV_1_ on the 6MWT are consistent with Eisner's study of younger patients (between 45 and 60 years) [3]. However, our results showed that the reduction in walked distance was slightly higher than the one found in the Eisner study (82.86 vs. 48.76 m). In addition to the physiological and psychological dysfunctions associated with age, one possible reason may be that the gait pattern has been altered since an increase in age yielded a reduction in the gait speed, stride length, and cadence in a study by Park et al [27].

### Limitations

Despite our findings, our study has several limitations. First, this study was restricted to a single region of Spain and most participants were at GOLD grade D; thus, our data may not be applicable to other settings and patients with other stages. Therefore, until further research is conducted using a broader patient cohort, these results should be interpreted with caution. Second, because our study population was relatively aging, our findings have limitations regarding the possible intervention for disability prevention. Third, we assessed isometric muscle strength with a hand-held dynamometer rather than a computerized isokinetic dynamometer, which can better isolation of muscle groups and more information about muscle dynamics. Nevertheless, hand-held dynamometers are more feasible and provide reliable and valid results, correlating strongly with isokinetic dynamometer results. We minimized measurement variability by having the same experienced physical therapist train the examiners in a standardized fashion and ensuring adequate inter-rater reliability [3, 28]. Fourth, we measure muscle strength on the dominant side as used previously by Eisner et al [3] in order to provide comparisons with their study population. However, dominance could have biased the relationship between muscle strength and FEV1. Fifth, the self-reported mobility questionnaire includes mobility activities from two ICF domains. It is unknown if more mobility activities from other ICF domains not assessed in the current study would be related to lung function. More research is needed in this areas.

Finally, although the results of the present study provide evidence that altered pulmonary function increases the risk of disability, the development of non-respiratory impairments and mobility limitations in body systems remote from the lung have a greater impact on disablement. Thus, the assessment and treatment of airway obstruction will not be sufficient to prevent the development of COPD-related disabilities [5]. In these subjects, exercise interventions aimed at improving peripheral muscle strength, physical endurance and mobility activities could potentially lower the subsequent risk of physical disability and restore normal functioning.

## Conclusion

This study provided evidence that pulmonary function impairment was related to the 6MWT and limitations on performance-based and self-reported mobility activities, but not skeletal muscle strength, among elderly COPD patients. In addition, pulmonary function had a greater impact on carrying lighter objects, walking up stairs, and walking in the neighbourhood. Pulmonary function measurements could be used for early screening of elderly COPD patients to identify those at higher risk of physical disability.

## Supporting information

S1 Table Database BDokPLOS_AGING COPD. (XLSX)
